# Constructing an ovarian cancer metastasis index by dissecting medical records

**DOI:** 10.18632/oncotarget.22336

**Published:** 2017-11-06

**Authors:** Yanjun Qu, Yanan He, Zhangming Li, Xiuwei Chen, Qian Liu, Shuangshuang Zou, Congcong Kong, Yixiu Liu, Ce Gao, Guangmei Zhang, Wenliang Zhu

**Affiliations:** ^1^ Department of Gynecology, The First Affiliated Hospital of Harbin Medical University, Harbin, China; ^2^ Department of Pharmacy, Guangdong Hospital of Integrated Chinese and Western Medicine, Foshan, China; ^3^ Department of Gynecology, The Third Affiliated Hospital of Harbin Medical University, Harbin, China; ^4^ Department of Obstetrics and Gynecology, The Second Affiliated Hospital of Harbin Medical University, Harbin, China; ^5^ Department of Pharmacy, The Second Affiliated Hospital of Harbin Medical University, Institute of Clinical Pharmacy, The Heilongjiang Key Laboratory of Drug Research, Harbin Medical University, Harbin, China

**Keywords:** ovarian cancer, metastasis, CA-125, model integration, ovarian cancer metastasis index

## Abstract

Globally, ovarian cancer (OC) is the leading cause of gynecological cancer-associated deaths. Metastasis, especially multi-organ metastasis, determines the speed of disease progression. A multicenter retrospective study was performed to identify the factors that drive metastasis, from medical records of 534 patients with OC. The average number of target organs per patient was 3.66, indicating multi-organ metastasis. The most common sites of metastasis were large intestine and greater omentum, which were prone to co-metastasis. Results indicated that ascites and laterality, rather than age and menopausal status, were the potential drivers for multi-organ metastasis. Cancer antigen (CA) 125 (CA-125) and nine other blood indicators were found to show a significant, but weak correlation with multi-organ metastasis. A neural network cascade-multiple linear regression hybrid model was built to create an ovarian cancer metastasis index (OCMI) by integration of six multi-organ metastasis drivers including CA-125, blood platelet count, lymphocytes percentage, prealbumin, ascites, and laterality. In an independent set of 267 OC medical records, OCMI showed a moderate correlation with multi-organ metastasis (Spearman ρ = 0.67), the value being 0.72 in premenopausal patients, and good performance in identifying multi-organ metastasis (area under the receiver operating characteristic curve = 0.832), implying a potential prognostic marker for OC.

## INTRODUCTION

Globally, close to 220,000 women develop ovarian cancer (OC) every year [1]. Owing to the lack of specific symptoms and effective markers for early detection [2, 3], most OC cases are diagnosed in their advanced stages. Compared to disease diagnosed at stage I or II, detection of OC at stage III or IV usually results in low 10-year survival rates of 21% and less than 5%, respectively [4]. OC is called as “the silent killer” and causes more than 100,000 gynecological cancer-associated deaths worldwide per year.

Recently, Cardenas et al. proposed that the origin and evolution of OC follow a four-step process that includes migration, seeding, induction, and expansion [5]. If the potential driving factors perturb the interaction between tumor cells, normal stromal cells, and the immune system, this process may be triggered [6]. According to the International Federation of Gynecology and Obstetrics (FIGO), the development of OC is divided into four stages [7]. At stage I, the tumor cells stay limited to the ovaries. With the homeostasis between tumor cells, normal stromal cells and the immune system is disturbed, tumor cells migrate to other pelvic organs (stage II), and beyond such as the abdomen and the lymph nodes (stage III), ultimately reaching outside of the peritoneal cavity (stage IV). The staging of OC by this method, suggests that the extent of tumor cell dissemination and growth determines the severity of the disease and the chances of survival [8].

Based on the pathological characteristics of OC as it progresses through the stages, we believe that monitoring tumor cell dissemination can help understand the exact status of the disease as well as help in its effective management. Unfortunately, there are not enough good biomarkers available for monitoring tumor cell dissemination and thereby the pathological progression of OC [9]. A clinically established serum biomarker is the cancer antigen (CA) 125 (CA-125) [10], a repeating peptide epitope of MUC16 [11]. CA-125 is a disease driver that gives impetus to the progression of OC by promoting tumor cell proliferation and inhibiting immune responses [12]. However, the use of CA-125 alone as a biomarker is not recommended due to its low sensitivity and limited specificity [13]. Attempts have been made to improve its performance by combining it with other biomarkers such as the human epididymis 4 (HE4) [14]. Recently, a combination of CA-125 and a panel of microRNAs have been suggested to be of potential value in monitoring progression of OC [15].

Based on the idea that routine medical examination indicators (MEIs) could be a potential source of biomarkers, we propose an alternative method for monitoring the progression of OC by making full use of the medical examination in clinical practice. Following the concept of CA-125-based combination biomarkers [14], an integration index can be made by considering multiple metastasis-related MEIs as inputs in a mathematical model. Compared with other potential biomarkers such as circulating microRNAs, MEIs have an obvious advantage of clinical availability [16]. This inherent advantage will help in using an MEI-based biomarker that is technically feasible in the current clinical context. In this study, an MEI-based biomarker was developed and validated by dissecting medical records of more than 800 OC patients. A function of the biomarker is to monitor the progression of OC and identify the risk of multi-organ metastasis. In summary, such an application will help facilitate disease management in OC.

## RESULTS

### Data collection from OC medical records and MEIs

**Figure 1:**
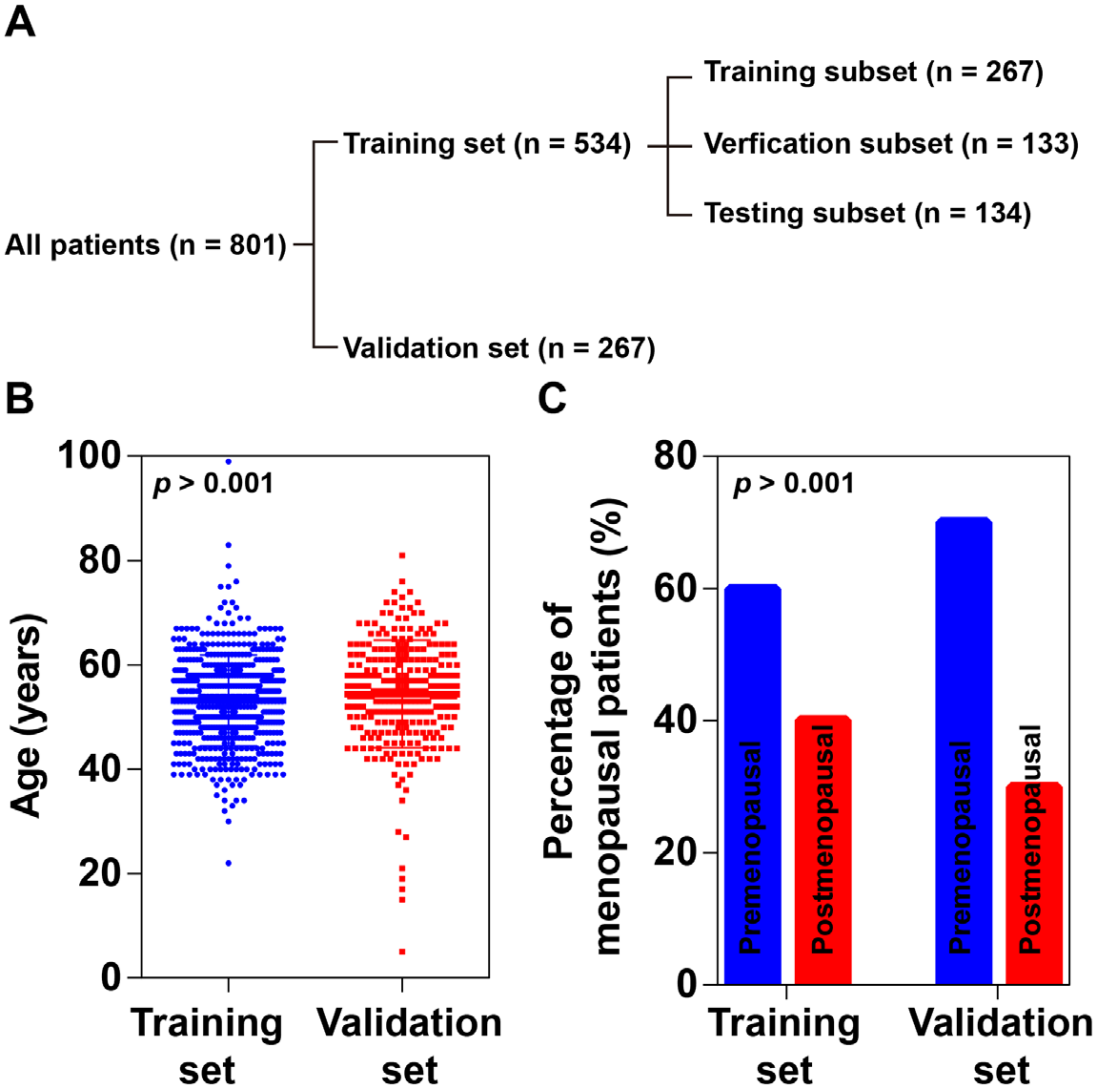
Patient grouping and demographic characteristics **(A)** A flowchart of patient grouping. **(B)** Age distribution of patients. No significant difference was found between the training set and validation set (P > 0.001, Student's *t*-test). **(C)** Proportions of postmenopausal patients. No significant difference was found between the training set and validation set (P > 0.001, Chi-square test).

In this study, 801 copies of OC medical records were collected from the affiliated hospitals of Harbin Medical University and were divided into two independent datasets including a training set and a validation set (Figure 1A). The training set contained 534 copies of medical records, from each of which 88 items of MEIs were collected (Supplementary Table 1). The validation set was constituted by 267 copies of medical records. Data of 23 items of MEIs were collected for each medical record in the validation set (Supplementary Table 2). All the MEIs were measured when patients were first diagnosed with OC. No significant difference was observed in age and proportion of postmenopausal patients between the training set and the validation set (Figure 1B and 1C).

### Epidemiological characteristics of metastasis in patients with OC

**Figure 2:**
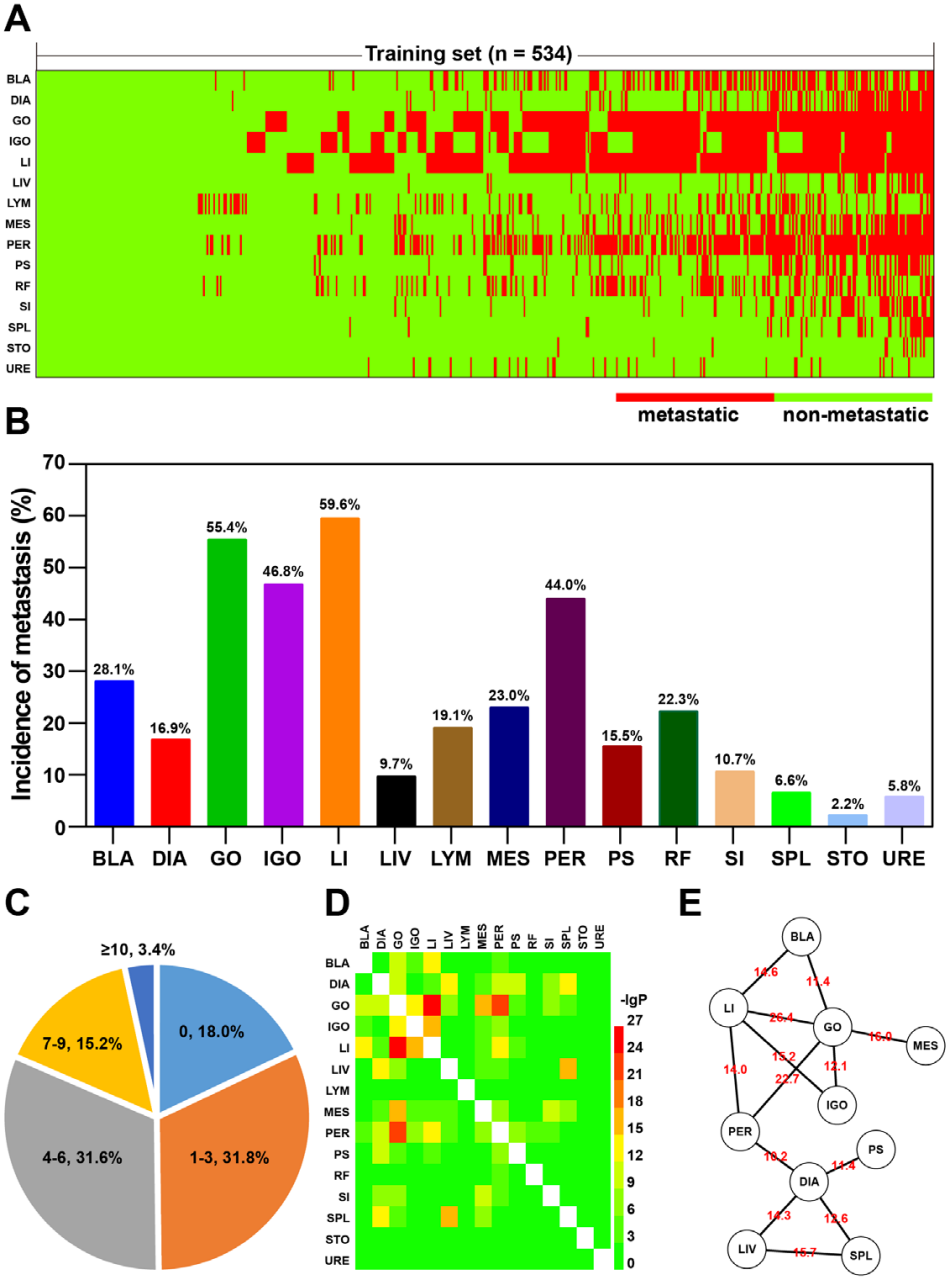
Metastasis tendencies in patients with OC **(A)** Heatmap of metastatic sites in a training set (n = 534). BLA: bladder; DIA: diaphragm; GO: greater omentum; IGO: internal genital organ; LI: large intestine; LIV: liver; LYM: lymph node; MES: mesentery; PS: paracolic sulci; PER: peritoneum; RF: rectouterine fossa; SI: small intestine; SPL: spleen; STO: stomach; URE: ureter. **(B)** Incidences of metastases at different sites. **(C)** Percentage pie chart of patients based on the number of metastatic sites. **(D)** Heatmap of adjusted hypergeometric probability (-lgP). **(E)** Network illustration of co-metastatic sites (-lgP > 10). The –lgP values are shown as edge attribute.

Briefly, one copy of a medical record corresponded to one patient who was clinically diagnosed with OC and underwent oophorectomy. A total of 15 possible metastatic sites were intraoperatively examined for each patient. Figure 2A presents an overview of all the metastasis in 534 patients of the training set. The highest incidence of metastases was observed for the large intestine (59.6%), followed by the greater omentum (55.4%) and the internal genital organ (46.8%) (Figure 2B). On the contrary, the stomach and the ureter were found to have a low incidence of metastasis, less than 6%. On an average, the number of metastatic sites was 3.66 per patient in the training set, implying multi-organ metastasis. It was found that half of the patients showed metastasis to more than three organs, and in 18% of the patients, no metastasis to any organ was observed (Figure 2C). Furthermore, by using the hypergeometric test, we found that some organs tended to be targeted for co-metastasis at a significant level (Figure 2D). One such example was the large intestine and the greater omentum, the two being targets of co-metastasis (Figure 2E). Another scenario was a patient showing simultaneous metastasis to both the spleen and the liver, despite the fact that the incidence of metastasis to these organs was relatively low (Figure 2B and 2E).

### Ascites and laterality were driving factors of multi-organ metastasis

**Figure 3:**
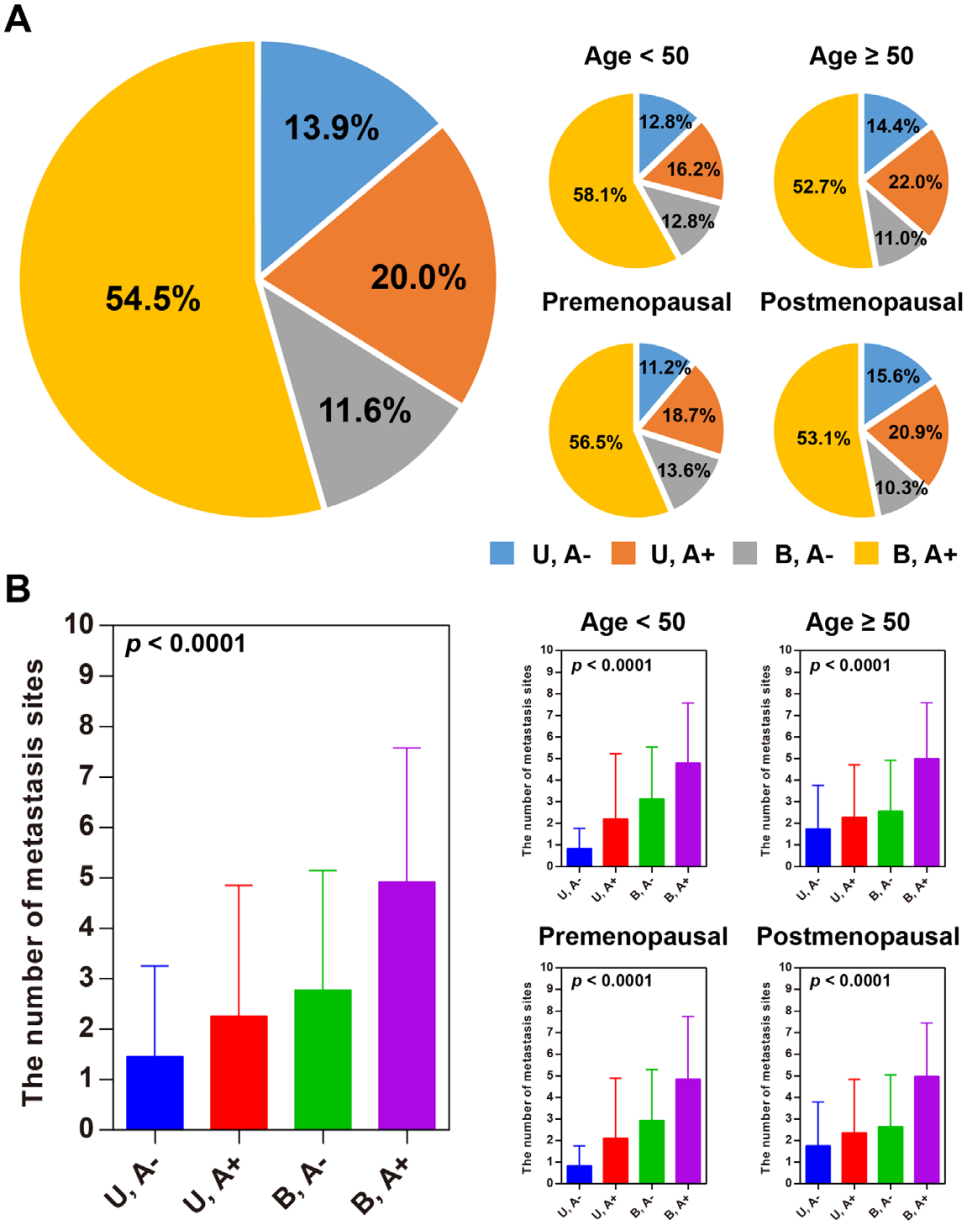
Ascites and laterality are related with multi-organ metastasis **(A)** Percentage pie charts of patients based on ascites and laterality. S: unilateral ovarian cancer; D: bilateral ovarian cancer; A-: without ascites; A+: with ascites. **(B)** Ascites and unilateral ovarian cancer are driving factors of multi-organ metastasis.

In the training set, about 55% of the patients developed ascites and bilateral ovarian cancer (Figure 3A), independent of age and menopausal status. Furthermore, we observed that bilateral OC patients with ascites were more inclined to develop multi-organ metastasis compared to unilateral OC patients without ascites (Figure 3B). This result suggested that ascites and laterality were synergistic drivers of multi-organ metastasis. Similar tendencies were observed in each of the subsets divided by age or menopausal status. This result implies that both age and menopausal status had a relatively weak impact on multi-organ metastasis. Ascites and laterality were found to significantly correlate with the number of metastatic sites in the training set (ρ = 0.329 for ascites and ρ = 0.451 for laterality)

### CA-125 and nine blood test MEIs were identified to be correlated with multi-organ metastasis

**Figure 4:**
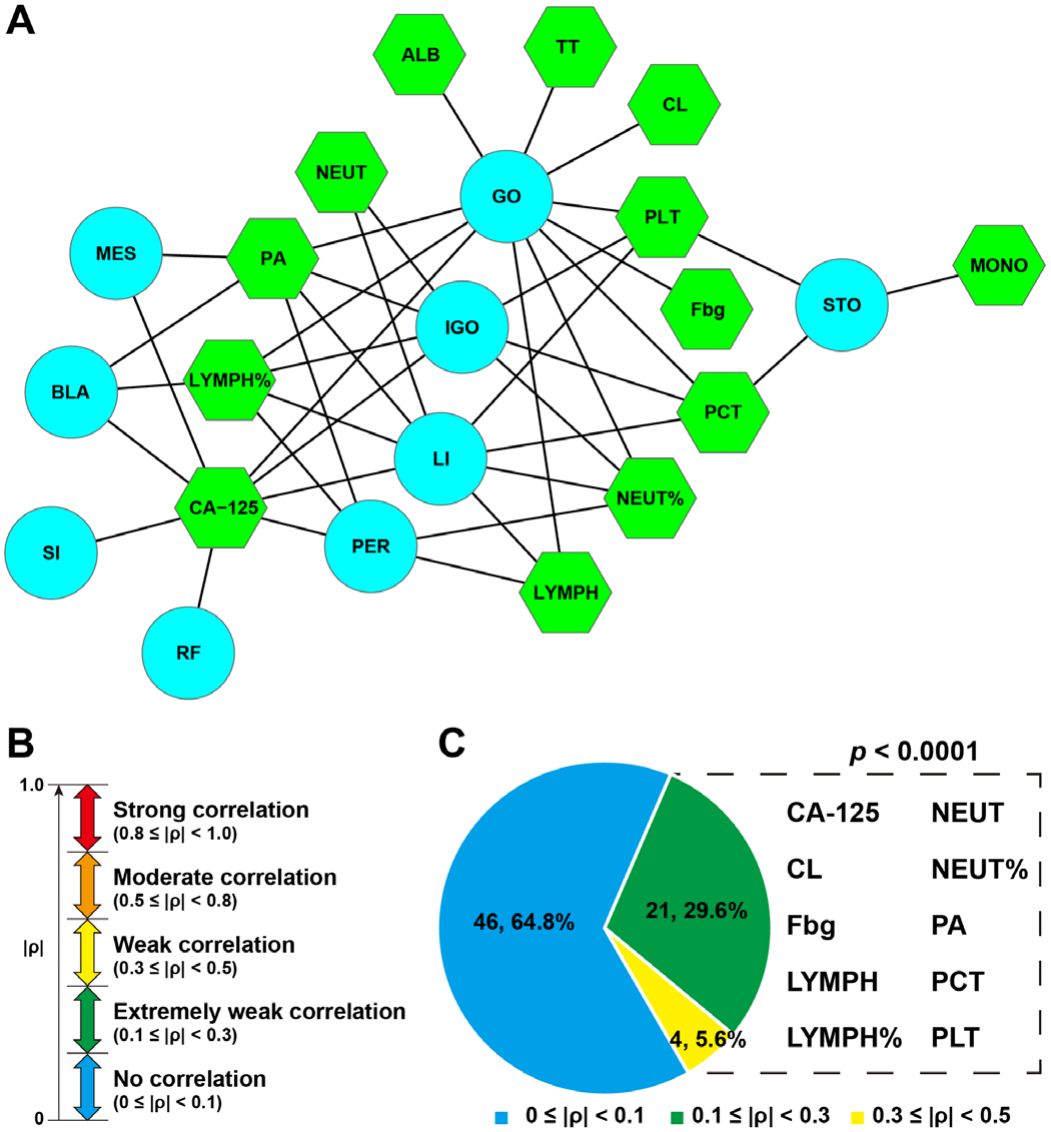
CA-125 and nine blood test indicators that were identified as MCM-related MEIs **(A)** A network of metastasis-related MEIs and metastatic sites. BLA: bladder; GO: greater omentum; IGO: internal genital organ; LI: large intestine; MES: mesentery; PER: peritoneum; RF: rectouterine fossa; SI: small intestine; STO: stomach. ALB: albumin; CL: chloridion; Fbg: fibrinogen; LYMPH: lymphocyte count; LYMPH%: percent lymphocytes; MONO: monocyte count; NEUT: neutrophil count; NEUT%: percent neutrophils; PA: prealbumin; PCT: thrombocytocrit; PLT: blood platelet count; TT: thrombin time. **(B)** Definition of correlation intensity. **(C)** MCM-related MEIs.

A receiver operating characteristic (ROC) curve test was performed to explore whether tumor biomarkers or blood/urine test indicators could identify the presence of metastasis in a given organ. Our results suggested that 13 MEIs were significantly associated with metastasis (P < 0.001, Figure 4A). Ten of them were found to correlate extremely weakly or weakly with the number of metastatic sites, suggesting that they were multi-organ metastasis (MOM)-related MEIs (Figure 4B and 4C).

### Integration of MOM-related MEIs by building a neural network cascade (NNC) model

**Figure 5:**
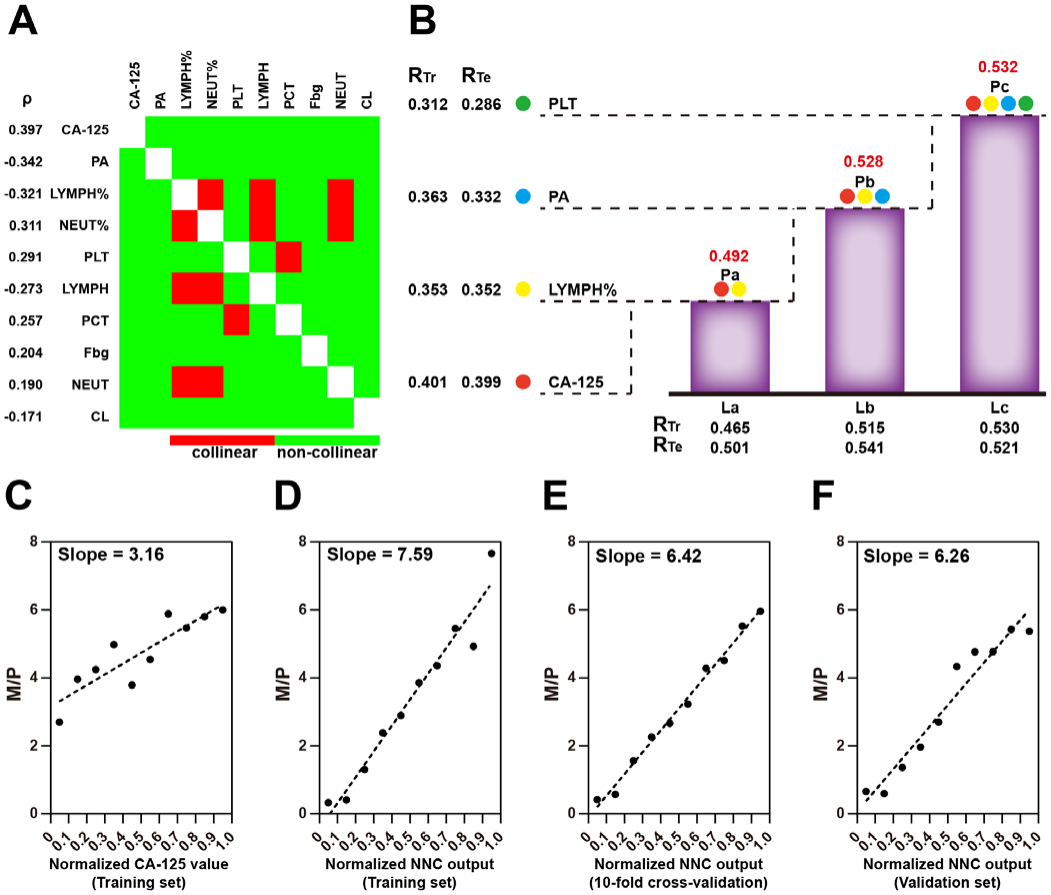
Model integration of MOM-related MEIs **(A)** Heatmap of MOM-related MEI co linearity. **(B)** NNC integration of MOM-related MEIs. La~Lc represent the ladder sub models in which the corresponding MEIs were imported; Pa~Pc are the integrated NNC parameters. For each sub model, RTr and RTe are shown. Spearman's rho indicates the correlation between the number of metastatic sites and the integrated NNC parameter. NEUT%: percent neutrophils; PA: prealbumin; PLT: blood platelet count. Scatter plots of M/P are illustrated as of normalized CA-125 values **(C)** and normalized NNC outputs **(D)**: training set; **(E)**: 10-fold cross-validation; **(F)**: validation set divided into 10 equidistant intervals, respectively.

As single MOM-related MEIs correlated extremely weakly or weakly with the number of metastatic sites, model integration was considered to obtain a stronger correlation between these two factors. Before the model integration, four MEIs were eliminated due to significant collinearity with other MEIs and a relatively low |ρ| (Figure 5A). Next, an NNC model was built for model integration of the four MEIs including CA-125, neutrophil percentage, prealbumin, and blood platelet count. Figure 5B illustrates the architecture of the NNC model. Chloridion and thrombin time were not integrated into the model because they did not contribute to enhancing the correlation between the NNC output and multi-organ metastasis. The NNC output was shown to have a stronger correlation with multi-organ metastasis than with CA-125 (ρ = 0.532 for the NNC output; ρ = 0.397 for CA-125). According to our cut off for correlation level (Figure 5B), the NNC output presented moderate correlation with the number of metastatic sites. A higher ρ led to a more inclined fit line making it easier to distinguish different patients, compared with the relatively low ρ of a single MOM-related MEI, such as CA-125 (Figure 5C and 5D). The 10-fold cross-validation and the independent external validation confirmed the effectiveness of the NNC model integration (Figure 5E and 5F).

### Establishment of ovarian cancer metastasis index (OCMI)

**Figure 6:**
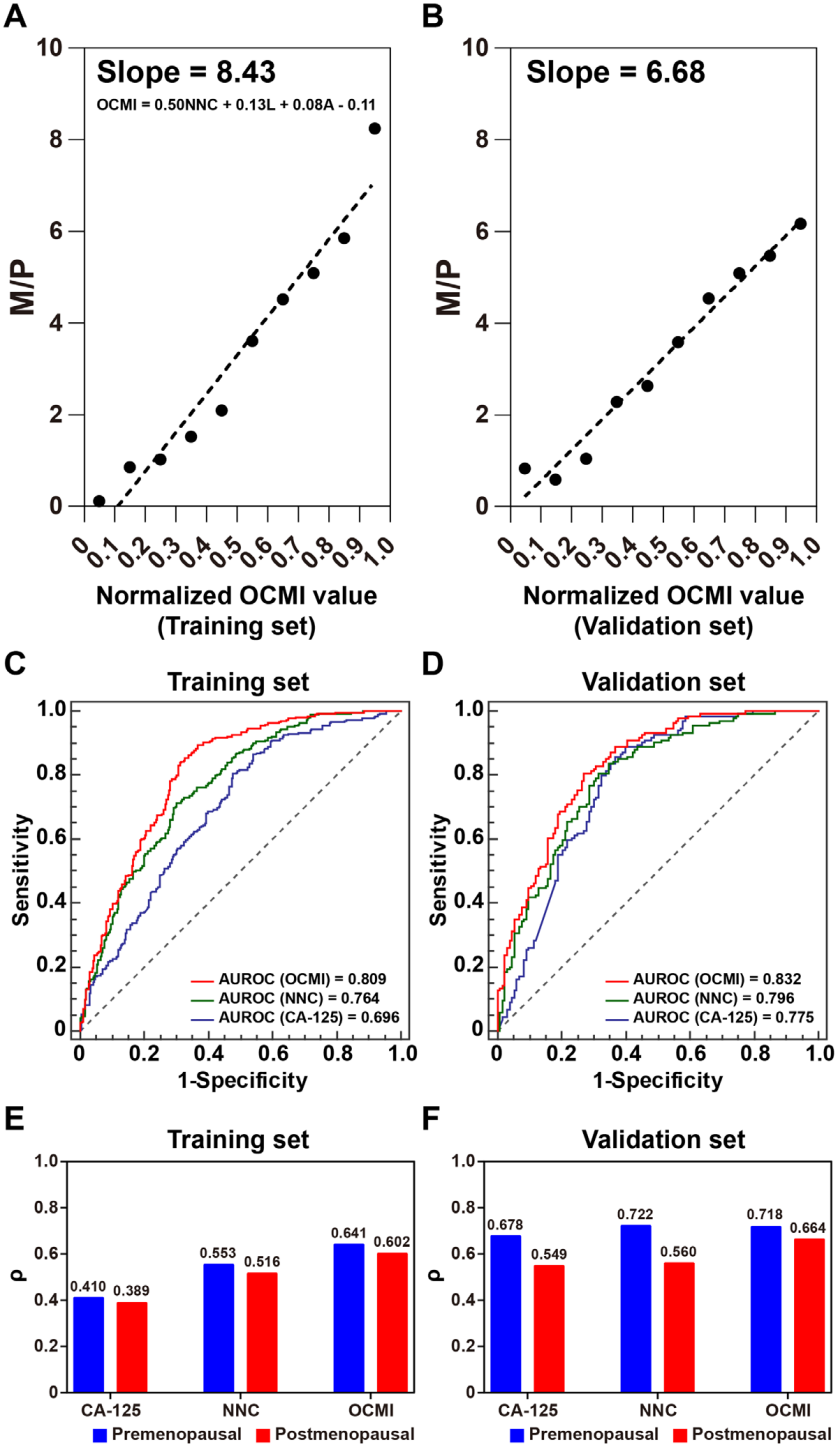
Performance assessment and validation of OCMI for identifying multi-organ metastasis Scatter plots of M/P are illustrated as normalized OCMI values **(A)**: training set; **(B)**: validation set were divided into 10 equidistant intervals. OCMI: ovarian cancer metastasis index; NNC: the output of the NNC model; L: Laterality (0: Unilateral/1: Bilateral) A: ascites (0: Not detected/1: Detected). ROC curves of CA-125, NNC output, and OCMI to identify multi-organ metastasis in the training set **(C)** and the validation set **(D)**. Multi-organ metastasis: the number of metastatic sites is more than 3. Column charts of Spearman’ rho (ρ) of patients in the training set **(E)** and the validation set **(F)** have been grouped by the menopausal state.

A multiple linear regression (MLR) model was used for further integrating NNC output, ascites, and laterality into a digital number that we named OCMI (Figure 6A). Compared with the NNC output, OCMI presented a stronger correlation with the number of metastatic sites (ρ = 0.617 for OCMI). A ρ of 0.673 was calculated between OCMI and the number of metastatic sites in the validation set. A moderate correlation between OCMI and the number of metastatic sites made it easier to distinguish between patients with a different number of metastatic sites. Compared with the NNC output, OCMI made it easier to differentiate the patients in the validation set (Figure 5F and 6B). ROC curve analysis results revealed that OCMI could more successfully identify multi-organ metastasis compared to CA-125 and NNC (Figure 6C and 6D). Our results suggested that, compared to CA-125 and NNC, OCMI presented the best performance correlating with the number of metastatic sites, in both premenopausal and postmenopausal women (Figure 6E and 6F), implying its potential application in predicting the risk of multi-organ metastasis in OC patients. Furthermore, OCMI was found to successfully distinguish patients without metastasis from those with metastasis with an area under the receiver operating characteristic (ROC) curve (AUC) of 0.879.

## DISCUSSION

Despite being the second most common gynecologic malignancy, OC causes the largest number of gynecological cancer-associated deaths in the world [17, 18]. The scarcity of good diagnostic and prognostic tools, failure to make an early diagnosis, rapid metastasis, and tumor heterogeneity all make the clinical management of OC difficult [19]. In the present study, we provided a new prognostic biomarker, OCMI, which was generated by integrating six MEIs, for identification of multi-organ metastasis. Rationality of creating OCMI was based on the clinical observation that multi-organ metastasis is the main cause of the high lethality of OC [20]. Our results suggest that OCMI exhibited superior performance in identifying high-risk patients with OC than CA-125 alone did.

The epidemiological observation in 534 patients with OC revealed that more than half of the patients had multi-organ metastasis, but merely less than one in five patients showed no metastasis to any of the fifteen organs when first diagnosed. This observation was in line with the proven finding that a large proportion of OC patients actually end up succumbing to non-OC cancer [20]. Widespread metastasis of tumor cells should be considered as a main epidemiological characteristic of OC. An obvious predilection in metastasizing OC tumor cells for large intestine and greater omentum was observed. Furthermore, our results suggest that co-metastasis to these two organs was a common event among patients with established OC. The presence of ascites was associated with this event as ascites were found in more than 90% of patients with metastasis to the large intestine and greater omentum.

Compared to other cancers, OC is the most common cause of the formation of malignant ascites [21]. Malignant ascites provides tumor cells with an optimal transfer station for further dissemination to distant organs [22]. Consistent with the above findings, ascites was identified as a significant indicator for multi-organ metastasis in this retrospective study. Compared to ascites, laterality was identified as another strong indicator for multi-organ metastasis. This finding was in line with a clinical report that about 60% of malignant OC was detected as bilateral ovarian carcinoma [23]. A reasonable inference is that bilaterality should be considered as two primary tumors rather than a consequence of collateral metastasis, considering the synergistic effect of bilaterality and ascites on promoting multi-organ metastasis [24].

It was verified that NNC-based integration of three blood MEIs markedly enhanced the power of CA-125 to identify high-risk patients. The three MEIs are neutrophil percentage, prealbumin, and blood platelet count. Neutrophils were experimentally validated to promote epithelial-to-mesenchymal transition and OC cell migration [25]. Different from the clinical findings of Paik and his colleagues [26], our results suggest that the relative indicator neutrophil percentage is superior to the absolute indicator neutrophil count to be a prognostic indicator of OC due to the stronger association with multi-organ metastasis. Low serum concentration of prealbumin represents malnutrition that is common in OC. Geisler et al. have demonstrated that significant mortality occurs in OC patients with relatively low serum concentrations of prealbumin [27]. Recent clinical surveys found that serum concentrations of prealbumin negatively correlate with levels of vascular endothelial growth factor and C-reactive protein, suggesting the connection between nutritional impairment and tumor metastasis and systemic inflammation in patients with OC [28, 29]. A positive correlation between blood platelet count and multi-organ metastasis was suggested by our study. This finding is in line with previous observations that elevated platelet count is a prognostic indicator of OC [30, 31]. Another supporting evidence is that functional interactions between platelets and OC cells play an important role in inducing epithelial-to-mesenchymal transition, invasion, and metastasis [32, 33].

As our previous work suggests [34–36], relying on a more complex model rather than using more input parameters is more effective in identifying the potential risk. In this study, a NNC-MLR hybrid model was established to construct the OCMI. NNC uses a ladder-like architecture of simple neural network elements. This makes it more complex than the traditional artificial neural network. In this study, ascites and laterality were examined intraoperatively rather than preoperatively. This is due to the objective limitation of the study. In the clinic, functional magnetic resonance imaging (MRI) is a preferred choice for diagnosis and evaluation of OC [37], and both ascites, as well as laterality, can be recognized as features from an MRI image. Ma et al. compared the features of ovarian clear cell carcinoma and high-grade serous carcinoma, which included ascites and laterality [38]. Despite the authors emphasizing the consistency of MRI and intraoperative examinations, no performance evaluation data such as accuracy was provided. In the present study, only 10% of the medical records contained MRI examination, and therefore, we could not assess the consistency of MRI and intraoperative examinations and use MRI-characterized ascites and laterality data for constructing OCMI. Further studies have to be performed to prove the value of MRI on the OCMI assessment of patients with OC.

Two potential limitations of the present study should be addressed. First, a small patient set was used to create OCMI. Despite an independent validation set being used to verify the effectiveness of NNC and OCMI in identifying high-risk patients, a larger patient set should be definitely tested for optimization of NNC and OCMI and for further assessment of their usefulness in OC management. Second, a correlation analysis between MEIs and multi-organ metastasis was performed on a limited number of MEIs, including age, menopausal status, CA-125, blood and urine test MEIs, ascites, and laterality. In the future study, clinical imaging examination and personal life habits questionnaire should be considered and investigated with equal importance.

In conclusion, we provide a new means to evaluate the risk of metastasis and the extent of multi-organ metastasis in patients with OC. Integration of only four to six routine medical indicators is sufficient to make NNC and OCMI more clinically feasible. As a metastasis identification indicator, OCMI is expected to make it possible to effectively evaluate and monitor the rate and direction of disease progression of OC in future clinical practice.

## MATERIALS AND METHODS

### Ethical statement

This study was approved by the Ethics Committee of the first affiliated hospitals of Harbin Medical University (Approval number: 201531) and was carried out in accordance with the Declaration of Helsinki.

### Access to medical records and MEIs collation

Electronic medical record systems of the second and third affiliated hospitals of Harbin Medical University were used to access the medical records of hospitalized patients who were clinically diagnosed with OC and had undergone oophorectomy. The MEIs included age, menopausal status, CA-125, blood and urine tests (68 items), ascites, laterality, and metastatic sites (n = 15). The 15 metastatic sites included bladder, diaphragm, greater omentum, internal genital organ, large intestine, liver, lymph node, mesentery, paracolic sulci, peritoneum, rectouterine fossa, small intestine, spleen, stomach, and ureter. All the medical records were divided into two independent datasets, a training set (n = 534, Supplementary Table 1) and a validation set (n = 267, Supplementary Table 2). The validation set used a truncated information collection strategy that excluded the blood and urine test items that were not significantly related to multi-organ metastasis.

### Metastasis tendency

In the training set, the percentage of incidence of metastases was calculated for each site, and a hypergeometric test was performed to evaluate the significance of co-metastasis among all the 15 studied sites. The hypergeometric probability was calculated as follows:

$$\text{P}=1-\overset{m}{\underset{k=0}{\sum }}\frac{\left(\begin{array}{c} n\\ k \end{array}\right)\left(\begin{array}{c} N-n\\ M-k \end{array}\right)}{\left(\begin{array}{c} N\\ M \end{array}\right)}$$

Where N is the total number of metastases involving two sites *i* and *j*, M is the total number of metastases to site *i*, n is the number of metastases to site *j*, m is the number of metastases to both sites. A total of 105 hypergeometric tests were performed for the 15 sites. To avoid false positives, all the P values were adjusted by using an EXCEL calculator of Holm-Bonferroni sequential correction developed by Gaetano J [39]. Co-metastasis was considered significant only if the adjusted P < 0.001 (–lgP > 3). HemI version 1.0.3.3 [40] and Cytoscape version 2.8.3 (Institute for Systems Biology, Seattle, WA, USA) [41] were applied to build a co-metastasis heatmap and a co-metastatic sites network by using the values of –lgP, respectively.

### Identification of cancer metastasis-related MEIs

GraphPad Prism version 6.0 (GraphPad Software, Inc., La Jolla, CA, USA) was used for identifying metastasis-related MEIs. First, a ROC curve test was performed to check whether a given MEI could identify single site cancer metastasis with an AUC of more than 0.5 and a P value of less than 0.001. To determine MEIs that were related with multi-organ metastasis, Spearman's correlation test was performed to evaluate the correlation between MEIs and the number of metastatic sites per patient. Spearman's correlation test was applied instead of Pearson's correlation test since some of the MEIs could not pass the D’Agostino-Pearson omnibus normality test and did not comply with the Gaussian distribution. A correlation was considered significant only if |ρ| (the absolute value of Spearman's rho) was more than 0.1 and P was less than 0.001. Besides, co-linearity among MEIs was checked and graphically displayed in a heatmap using HemI version 1.0.3.3. Co-linearity was determined to be present if a |ρ| of > 0.3 was calculated for any two MEIs.

### Integration of MOM-related MEIs

All of the MOM-related MEIs were normalized into a 0 to 1 digital number for data unification as previously described [34]. Following the previously established procedure [35], an NNC model was built for integration of the MOM-related MEIs by using the normalized number of metastatic sites as a model output. The Intelligent Problem Solver (IPS) tool in the software STATISTICA Neural Networks (SNN, Release 4.0E; Statsoft, Tulsa, OK, USA) was applied to construct 1-11-1 or 2-11-1 radial basis function (RBF)-ANN sub-models, which constituted the overall framework of the NNC. In IPS, all the medical records in the training set (n = 534) were randomly divided into three subsets (training subset, verification subset, and testing subset) in a 2:1:1 ratio and the holdout cross-validation method was built-in for preliminary model validation. The medical records in the testing subset (n = 133) did not participate in model building and were used for model testing. IPS calculated correlation coefficients for the training subset (R_Tr_) and the testing subset (R_Te_). The two correlation coefficients measured the correlation between the predicted and the actual numbers of metastatic sites of a patient. Similar values of R_Tr_ and R_Te_ indicate good generalization ability of the model. Finally, the SPSS statistical software version 19.0 (IBM Corp., New York City, NY, USA) was used to build an MLR model to further integrate NNC output and the two intraoperative examination items, ascites and laterality, into a digital parameter, OCMI.

### Model validation and performance evaluation

For the NNC model, the 10-fold cross-validation method was used for model validation as previously described [35]. Briefly, all the medical records were randomly divided into 10 mutually exclusive sets of nearly equal size. Next, nine were selected for model training, and one was used for model validation. The above procedure was repeated 10 times to allow each of the 10 medical records sets to be independently used for validation. Spearman's correlation test was used to investigate the significance and the level of correlation between the predicted and the actual numbers of metastatic sites in a patient. For both the NNC model and the MLR model, the validation set (n = 267, Supplementary Table 2) was used for external validation. The Spearman's rho and slope calculations were applied for performance evaluation of the two models. A slope was calculated from the scatter plot of M/P in 10 equidistant intervals of normalized CA-125/NNC output/OCMI. M/P is the ratio of the sum of metastatic sites divided by the sum of patients in an interval.

### Data statistics

Data are presented as mean ± standard deviation (SD). GraphPad Prism version 6.0 was applied to conduct the following statistical analyses, including a ROC curve test, D’Agostino-Pearson omnibus normality test, Spearman's correlation test, Student's *t*-test, Chi-square test, one-way ANOVA test, and Holm-Sidak's multiple comparisons test. In this study, differences were considered as statistically significant when P < 0.001.

## SUPPLEMENTARY MATERIALS FIGURES AND TABLES







## Abbreviations

OC: ovarian cancer
OCMI: ovarian cancer metastasis index
CA-125: cancer antigen (CA) 125
MEI: medical examination indicator
ROC: receiver operating characteristic
NNC: neural network cascade
AUC: area under the ROC curve
MLR: multiple linear regression
MRI: magnetic resonance imaging
