# Supplementary material for: Constructing an ovarian cancer metastasis index by dissecting medical records
Source: Oncotarget. 2017 Nov 6;8(60):102212–22. doi: 10.18632/oncotarget.22336 (PMC5731947; doi:10.18632/oncotarget.22336)
Supplement: Supplementary file 1 [file oncotarget-08-102212-s001.pdf]

Constructing an ovarian cancer metastasis index by dissecting medical records

SUPPLEMENTARY MATERIALS

Supplementary Table 1: Metastatic site and clinical examination results for the patients included in this study (Training set)

See Supplementary Table 1

Supplementary Table 2: Metastatic site and clinical examination results for the patients included in this study (Validation set)

See Supplementary Table 2
